# *Candida albicans* β-Glucan Differentiates Human Monocytes Into a Specific Subset of Macrophages

**DOI:** 10.3389/fimmu.2018.02818

**Published:** 2018-11-30

**Authors:** Julia Leonhardt, Silke Große, Christian Marx, Fatina Siwczak, Sven Stengel, Tony Bruns, Reinhard Bauer, Michael Kiehntopf, David L. Williams, Zhao-Qi Wang, Alexander S. Mosig, Sebastian Weis, Michael Bauer, Regine Heller

**Affiliations:** ^1^Center for Sepsis Control and Care, Jena University Hospital, Jena, Germany; ^2^Department of Anesthesiology and Intensive Care Medicine, Jena University Hospital, Jena, Germany; ^3^Institute of Molecular Cell Biology, Center for Molecular Biomedicine, Jena University Hospital, Jena, Germany; ^4^Leibniz Institute on Aging, Fritz Lipmann Institute, Jena, Germany; ^5^Institute of Biochemistry II, Jena University Hospital, Jena, Germany; ^6^Department of Internal Medicine IV, Jena University Hospital, Jena, Germany; ^7^Institute of Clinical Chemistry and Laboratory Diagnostics Jena University Hospital, Jena, Germany; ^8^Department of Surgery and Center of Excellence in Inflammation, Infectious Disease and Immunity, Quillen College of Medicine, East Tennessee State University, Johnson City, TN, United States; ^9^Center for Infectious Diseases and Infection Control, Jena University Hospital, Jena, Germany

**Keywords:** β-glucan, *Candida albicans*, monocyte survival, monocyte to macrophage differentiation, trained immunity

## Abstract

β-Glucan derived from cell walls of *Candida albicans* is a potent immune modulator. It has been shown to induce trained immunity in monocytes via epigenetic and metabolic reprogramming and to protect from lethal sepsis if applied prior to infection. Since β-glucan-trained monocytes have not been classified within the system of mononuclear phagocytes we analyzed these cells metabolically, phenotypically and functionally with a focus on monocyte-to-macrophage differentiation and compared them with naïve monocytes and other types of monocyte-derived cells such as classically (M1) or alternatively (M2) activated macrophages and monocyte-derived dendritic cells (moDCs). We show that β-glucan inhibits spontaneous apoptosis of monocytes independent from autocrine or paracrine M-CSF release and stimulates monocyte differentiation into macrophages. β-Glucan-differentiated macrophages exhibit increased cell size and granularity and enhanced metabolic activity when compared to naïve monocytes. Although β-glucan-primed cells expressed markers of alternative activation and secreted higher levels of IL-10 after lipopolysaccharide (LPS), their capability to release pro-inflammatory cytokines and to kill bacteria was unaffected. Our data demonstrate that β-glucan priming induces a population of immune competent long-lived monocyte-derived macrophages that may be involved in immunoregulatory processes.

## Introduction

*Candida albicans* β-1-3,1-6-glucan (β-glucan), a pathogen-associated molecular pattern (PAMP) present in the fungal cell wall, has been characterized as a potent immune modulator. It has been shown to mediate a phenomenon termed trained (innate) immunity, which describes the ability of innate immune cells to react with an enhanced immune response after a first pathogen insult (1). In contrast to the immune memory mediated by the adaptive immune system, which is the basis for vaccination, innate immune memory has only been described recently and has been shown to involve immune cells such as myeloid progenitors, natural killer cells, and monocytes (2–5). β-Glucan is the best characterized stimulus to induce trained immunity in monocytes. It has been shown to trigger epigenetic remodeling and metabolic reprogramming through a pathway involving dectin-1, the surface receptor of β-glucan, and the PI3K/Akt/mTOR (phosphoinositide 3-kinase/Akt/mechanistic target of rapamycin) signaling cascade (6, 7). Transient treatment of myeloid cells with β-glucan has been reported to protect mice from subsequent sepsis (6).

Since β-glucan-induced trained immunity is a promising prophylactic therapy for patients prone to infections (e.g., patients undergoing major elective surgery), a complete understanding of the underlying processes is pivotal. So far, the classification of trained monocytes remains enigmatic (8). This is underlined by the heterogeneous terminology, referring to β-glucan-trained cells as “trained monocytes” (6, 9), “memory macrophages” (8), “trained macrophages” (7, 10) or “circulating differentiated monocytes” (4). The current study was designed to characterize effects of β-glucan on monocyte differentiation. β-Glucan-treated monocytes were compared with classically (M1-like) and alternatively activated (M2-like) monocyte-derived macrophages and monocyte-derived dendritic cells (moDCs) with respect to metabolism, phenotype and function. Our data show that β-glucan protects monocytes from spontaneous apoptosis and promotes differentiation into a specific subset of metabolically highly active macrophages, which exhibit an M2-like surface marker profile. β-Glucan-differentiated macrophages are able to kill live bacteria and to respond to LPS with secretion of proinflammatory cytokines and with an increased release of IL-10.

## Methods

### Isolation and culture of human monocytes

Peripheral blood was collected from healthy, male, non-smoking volunteers after obtaining informed consent and approval by the Institutional Ethics Committee. Blood mononuclear cells (PBMCs) were isolated using density gradient centrifugation (Biocoll, Merck Millipore). Classical monocytes (CD14^++^ CD16^−^) were purified by negative selection (Dynabeads Untouched Human Monocytes Kit, Thermo Fisher Scientific). High purity and viability (both ≥ 90%) of isolated cells were confirmed by flow-cytometric detection of CD14 expression and propidium iodide (PI)/annexin V staining, respectively. Freshly prepared monocytes were seeded at a density of 3 × 10^5^ cell/cm^2^ and incubated in RPMI 1640 medium (Dutch modification, Sigma-Aldrich) including 100 μg/ml gentamicin, 1 mM sodium pyruvate (Thermo Fisher Scientific), 2 mM GlutaMAX™ (Thermo Fisher Scientific) and 10% heat-inactivated human AB serum (Sigma-Aldrich) at 37°C and 5% CO_2_. Medium was refreshed after 3 days.

### Stimulation of monocytes

One hour after isolation, cells were stimulated with β-glucan extracted from yeast *C. albicans* (5 μg/ml or 50 μg/ml) or macrophage colony-stimulating factor (M-CSF, 50 ng/ml, Peprotech) for 24 or 48 h or left untreated (control). After β-glucan treatment for 24 h (priming), cells were gently washed and incubated for up to another 6 days. Time points for analysis of survival, growth factor release, metabolism and surface markers in β-glucan-stimulated cells are detailed below.

### *In vitro* generation of M1, M2, and modcs

Differentiation of monocytes into M1-like macrophages was performed by cultivation with 500 U/ml granulocyte-macrophage colony-stimulating factor (GM-CSF, Peprotech) for 7 days plus 100 ng/ml LPS and 20 ng/ml IFNγ (Peprotech) for the last 24 h. M2-like macrophages were obtained by applying 50 ng/ml M-CSF for 7 days plus 50 ng/ml IL-4 (Peprotech) for the last 24 h. For differentiation of monocytes into immature dendritic cells, 1,000 U/ml GM-CSF plus 50 ng/ml IL-4 was given for 7 days, while mature dendritic cells were generated by addition of 1,000 U/ml GM-CSF and 50 ng/ml IL-4 for 7 days plus activation with 100 ng/ml LPS for the last 24 h.

### Flow cytometry

To allow complete and gentle detachment of monocyte-derived cells, cell culture for flow cytometry experiments (viability, phenotyping) was performed on thermo-responsive plates (UpCell™ Nunc™, Thermo Fisher Scientific). For the analysis of viability, cells were stimulated for 24 or 48 h with β-glucan or M-CSF or left untreated. For phenotyping, monocytes of the same donor were either stimulated with β-glucan for 24 h followed by a resting period of 6 days or differentiated for 7 days into M1, M2 or moDCs as described above. After experimental treatments, cells were gently detached by incubating thermo-responsive plates at 4°C for 20 to 40 min. Flow-cytometric measurements were performed with the FACS Canto cytometer (BD Biosciences) or CytoFLEX (Beckman Coulter) using the following fluorochrome-labeled monoclonal antibodies and dyes: Apoptosis Detection Kit I including anti-annexin V-FITC and PI (BD Biosciences), anti-CD1a-PE (clone HI149, Biolegend), anti-CD11c-APC (clone S-HCL-3, BD Biosciences), anti-CD14-FITC (clone M5E2, BD Biosciences), anti-CD14-PE (clone TÜK4, Miltenyi Biotec), anti-CD14-PE/Cy7 (clone HCD14, Biolegend), anti-CD40-APC (clone HI40a, Immunostep), anti-CD83-APC (clone HB15e, Biolegend), anti-CD86-PE (clone IT2.2, Biolegend), anti-CD197(CCR7)-APC (clone G043H7, Biolegend), anti-CD206-FITC (clone 19.2, BD Biosciences), anti-FcεR1-FITC (clone AER-37, Biolegend), and anti-HLA DR-FITC (clone L243, Biolegend) and Horizon™ Fixable Viability Stain (FVS, BD Biosciences).

### Analysis of cell number

Cells were seeded on 24-well plates for cell counting and protein determination and on 96-well cell culture plates for measuring DNA content. After β-glucan priming for 24 h, cells were washed and kept in culture for 6 more days. Then, cells were counted in 16 defined high-power fields (9,400 μm2) using phase contrast microscopy and the mean cell number per high-power field was calculated for each experimental condition.

### Growth factor and cytokine measurements

Monocytes were seeded into 24-well plates and stimulated with β-glucan for 24 h. Thereafter, growth factors were analyzed in supernatants by means of a cytometric bead array applying the LEGENDplex Human Growth Factor Panel (Biolegend). Alternatively, cells were gently washed and allowed to rest for another 5 days. On day 6, medium was replaced by medium ± 10 ng/ml LPS (Sigma-Aldrich). On day 7, cytokines from supernatants were quantified by cytometric bead array using the Human Inflammatory Cytokines Kit (BD Biosciences). Results were normalized to the cell protein mass per well. To quantify cellular proteins, cells were lysed with buffer containing 0.1 M NaOH, 1% Na_2_CO_3_ and 1% SDS and the DC^TM^ Protein Assay kit (Bio-Rad) was applied.

### Immunoblotting

Cells were seeded into 30-mm culture dishes and incubated with the M-CSF inhibitor GW2580 (Sigma-Aldrich) for 1 h if indicated. Subsequently, monocytes were incubated with β-glucan or M-CSF for 24 or 48 h, then washed, lysed and immunoblotted as previously described (11). Briefly, lysates were separated by SDS-PAGE and transferred to PVDF membranes, which were blocked and incubated overnight at 4°C with primary antibodies [anti-cleaved Poly (ADP-ribose) polymerase (PARP), anti-PARP, anti-cleaved caspase 3, anti-caspase 3 (all from Cell Signaling)] diluted in TBST [20 mmol/l Tris (pH 7.6), 137 mmol/l NaCl, 0.1 % (v/v) Tween-20] containing 5% bovine serum albumin. After incubation with horseradish peroxidase-conjugated secondary antibodies for 1 h, signal detection was accomplished using enhanced chemiluminescence reagent (ECL^TM^). Protein bands were quantified by densitometry using ImageJ software.

### Immunohistochemistry

For microscopic analysis of morphology, monocytes were seeded into 24-well plates and stimulated for 24 h with β-glucan followed by a 6-day resting period as described above. On day 7 after seeding, cells were stained with May-Grünwald-Giemsa (Morphisto) and analyzed by light microscopy (Axio Observer Z1, Carl Zeiss).

### Metabolite analysis

For analysis of lactate production, monocytes were seeded into 24-well plates and stimulated for 24 h with β-glucan followed by a 5-day resting period as described above. On day 6, culture medium was replaced by medium ± 10 ng/ml LPS (Sigma-Aldrich). 24 h later, lactate concentration in supernatants was measured by an automated analyzer (Abbott Architect ci16200 integrated system, Abbott Laboratories).

### Metabolic profiling

Metabolic profiling of cells was performed using the Seahorse XF96 extracellular flux analyzer with a Cell Mito Stress Test (Agilent technologies). Monocyte-derived cells detached from thermo-responsive plates (UpCell™ Nunc™, Thermo Fisher Scientific) after different treatments or freshly isolated monocytes from the same donor were seeded into 96-well Seahorse XF96 microplates. 1 h (monocytes) or 24 h (monocyte-derived cells) after seeding, cell supernatants were replaced by Agilent Seahorse XF Assay Medium, pH 7.4, supplemented with 10 mM D-glucose (Sigma Aldrich) and 1 mM sodium pyruvate (Thermo Fisher Scientific). Cells were then cultured for another hour in a CO_2_-free incubator at 37°C. Oxygen consumption rate (OCR) and extracellular acidification rate (ECAR) were monitored at basal conditions and after sequential injections of 2 μM oligomycin (Abcam) to block the mitochondrial ATP synthase, 25 μM 2,4-dinitrophenol (2,4-DNP, Sigma Aldrich) to uncouple mitochondrial respiration from ATP production and 2 μM antimycin A (Sigma Aldrich) to fully inhibit mitochondrial respiration. Measurements were performed for 1,5 h in 3 min mix and 3 min measure cycles at 37°C in six replicates per condition. OCR and ECAR were depicted as pmoles/min and mpH/min, respectively, and normalized to the DNA content of each well, as described before (12). DNA was measured using the CyQuant assay (Thermo Fisher Scientific).

### Bacterial killing activity

Assays to analyze bacterial clearance were performed using a *Staphylococcus aureus* (*S. aureus*) strain USA300, which constitutively expresses high levels of green fluorescent protein (GFP) (kindly provided by Bas G. J. Surewaard, University of Calgary, Canada). Overnight cultures of bacteria were grown in Mueller-Hinton-broth (Sigma Aldrich), harvested by centrifugation, washed and resuspended in PBS at an OD_600nm_ 1.0, (CFU ~ 6 × 10^8^/ml). Freshly isolated monocytes were seeded into thermo-responsive plates (UpCell™ Nunc™, Thermo Fisher Scientific) and transiently stimulated for 24 h with 5 μg/ml β-glucan or left untreated (control). After washing and a resting period, cells were gently detached on day 6 and reseeded at equal densities into 6-well plates. On day 7, cells were counted in 7 defined high-power fields using phase contrast microscopy and total cell number per well was determined. Subsequently, cells were infected with live *S. aureus* at a MOI of 5. Infection and phagocytosis were controlled by fluorescence microscopy. After 90 min, supernatants were collected for counting the surviving extracellular bacteria. To determine the intracellular bacterial load, macrophages were incubated for further 30 min with 20 μg/ml lysostaphin (Bionexus), washed and lysed in sterile water. Bacteria were quantified after serial dilutions as colony forming units (CFU) on blood agar plates. The numbers of surviving intra- and extracellular bacteria were expressed as percentage of the original inoculum. The killing activity was calculated as difference between original inoculum and the surviving intra- and extracellular bacteria in % of the former.

### Statistical analysis

The statistical analysis was performed using SigmaPlot Software (San Jose, USA). Data are presented as mean ± SEM. Comparisons between multiple groups were made with one-way or two-way analysis of variance, if appropriate. If normality test failed, Kruskal-Wallis one-way analysis of variance on ranks was used. *Post hoc* comparisons were made with the Holm-Sidak test or the Dunn's method was used. Comparisons between two groups were made with Student's *t*-test for paired samples or Wilcoxon signed-rank test using Bonferroni correction for multiple uses, if appropriate. A *p*-value of < 0.05 was considered to be statistically significant.

## Results

### β-glucan inhibits spontaneous human monocyte apoptosis

**Figure 1:**
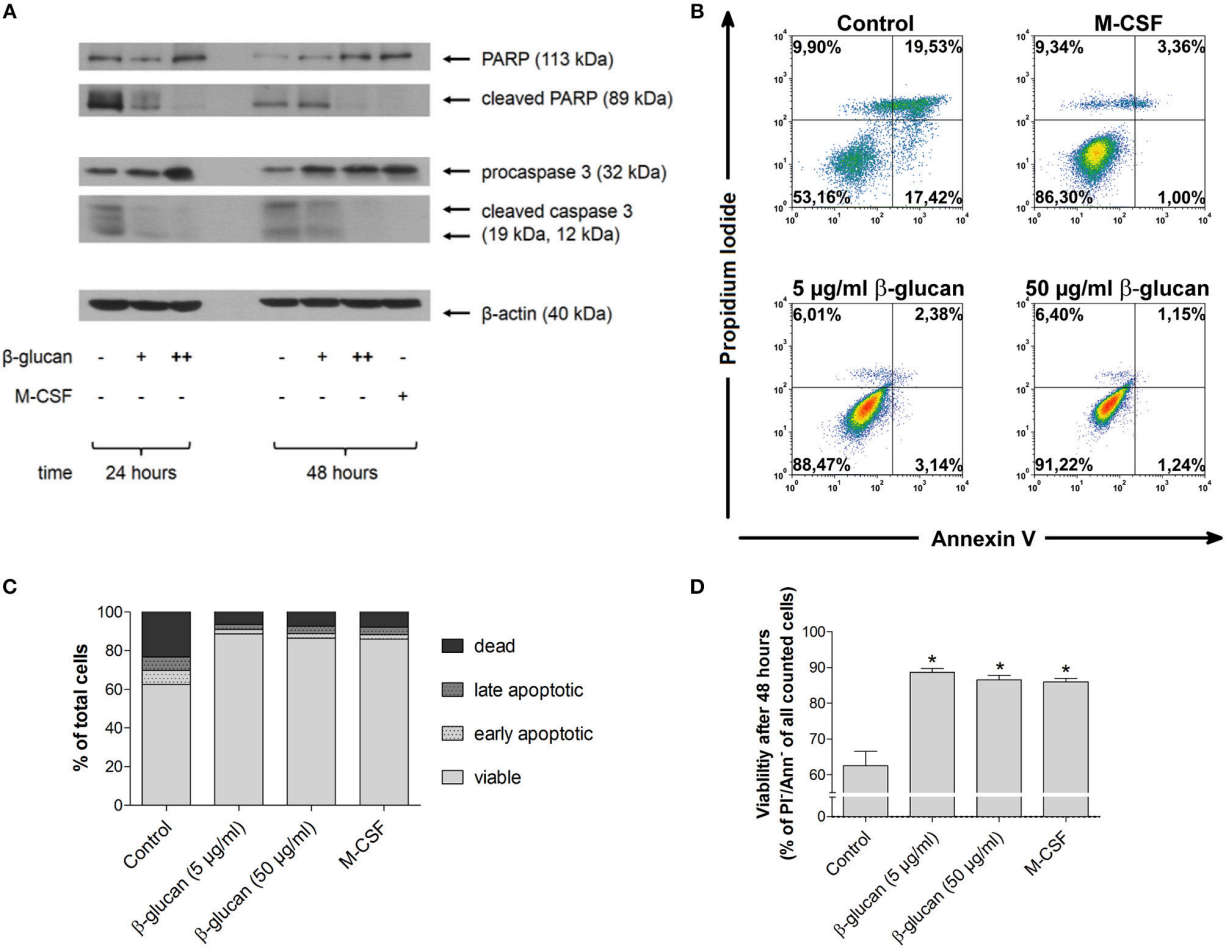
β-Glucan inhibits spontaneous human monocyte apoptosis. **(A)** Human monocytes were left untreated (control) or stimulated with 5 μg/ml β-glucan (+), 50 μg/ml β-glucan (++) or M-CSF (50 ng/ml). After 24 and 48 h, protein extracts were analyzed for cleavage of PARP and caspase 3. One representative of two (24 h) or three (48 h) independent experiments is shown. Densitometry data are given in the text. **(B–D)** Cell viability was measured after 48 h by flow cytometric analysis of Annexin V (Ann) and propidium iodide (PI). One representative dot plot is shown in **(B)** and the frequencies of dead (PI+/Ann-), late apoptotic (PI+/Ann+), early apoptotic (PI-/Ann+) and viable cells (PI-/Ann-) from five independent experiments are shown in **(C, D)**. The frequency of viable cells was significantly higher in the groups treated with M-CSF or β-glucan as compared to untreated monocytes (control) **(D)**. Values are means ± SEM, ^*^p < 0.05 compared to control.

In line with literature data (13, 14), monocytes underwent rapid spontaneous apoptosis if they were cultured in the absence of specific differentiation stimuli (control). This was demonstrated by caspase 3 activation and PARP inactivation after 24 and 48 h as well as by decreased viability after 48 h as analyzed by PI and annexin V staining (Figures 1A–D). In the presence of M-CSF spontaneous apoptosis was almost completely prevented (Figures 1A–D). Similarly, incubation of monocytes with β-glucan inhibited caspase 3 and PARP cleavage significantly in a dose-dependent manner. Densitometry analysis of protein bands revealed inhibition of PARP cleavage by 29.9 ± 15.5% or 71.3 ± 5.8% and inhibition of caspase cleavage by 48.3 ± 11.5% or 82.0 ± 3.4% when cells were treated for 48 h with 5 or 50 μg/ml β-glucan, respectively (*p* < 0.05 for all inhibitions). In line with inhibition of early apoptotic events, treatment of monocytes with β-glucan significantly enhanced cell viability after 48 h from 62.5% to more than 85% (Figures 1B–D).

### The anti-apoptotic effect of β-glucan is independent of M-CSF

**Figure 2:**
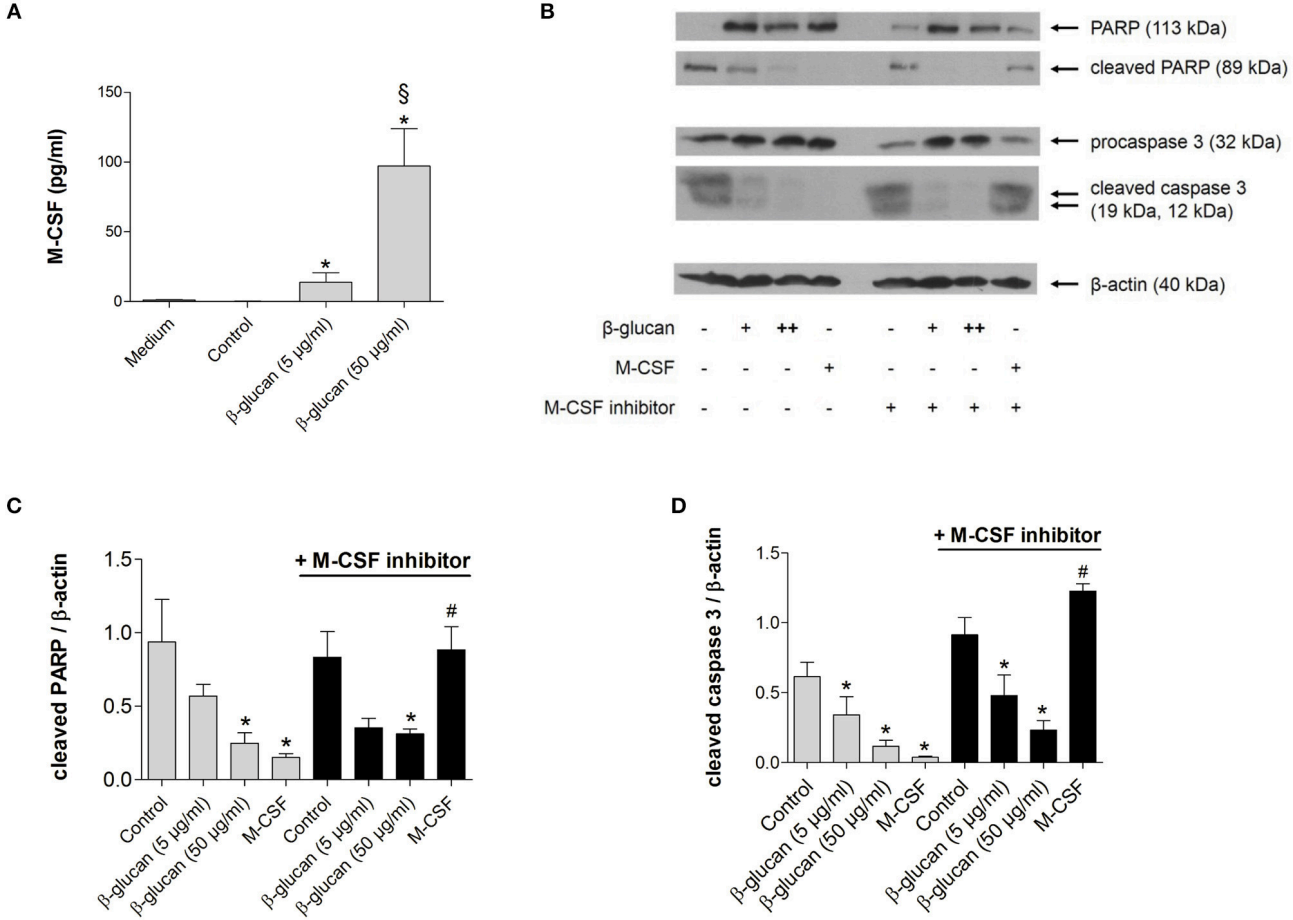
The anti-apoptotic effect of β-glucan is independent of M-CSF. **(A)** Human monocytes were left untreated (control) or stimulated for 24 h with β-glucan (5 μg/ml or 50 μg/ml). The release of M-CSF was determined by cytometric bead array in cell supernatants or in medium plus 10% serum only (medium). **(B–D)** Human monocytes were preincubated with the M-CSF inhibitor GW2580 or vehicle for 60 min and then incubated for 48 h with either M-CSF (50 ng/ml) or β-glucan (5 μg/ml or 50 μg/ml). Controls were left without stimulation. Protein lysates were analyzed in immunoblots for cleavage of PARP and caspase 3. **(B)** One representative blot is shown. **(C,D)** Densitometry analyses of protein bands normalized to β-actin of three independent experiments. Values are means ± SEM, ^*^p < 0.05 compared to the respective untreated control, §*p* < 0.05 compared to 5 μg/ml β-glucan, *#p* < 0.05 compared to M-CSF alone.

To investigate whether the anti-apoptotic effect of β-glucan was mediated by the autocrine or paracrine release of growth factors, we first analyzed whether M-CSF and GM-CSF were secreted by monocytes after 24 h of treatment with β-glucan. In control medium supplemented with 10% serum both growth factors were negligable. Treatment with β-glucan led to significant secretion of M-CSF (Figure 2A), while no significant GM-CSF release could be detected (data not shown). To analyze whether the M-CSF released by β-glucan-treated cells mediates the observed anti-apoptotic effects, we employed GW2580, a potent inhibitor of the M-CSF receptor. GW2580 (10 μM, 1h) completely prevented the anti-apoptotic effect of M-CSF as monitored by PARP and caspase 3 cleavage but had no effect on β-glucan-induced inhibition of apoptosis (Figures 2B–D). These data indicate that M-CSF is not involved in the anti-apoptotic effects induced by β-glucan.

### Transient treatment with β-glucan increases long-term survival of monocytes

**Figure 3:**
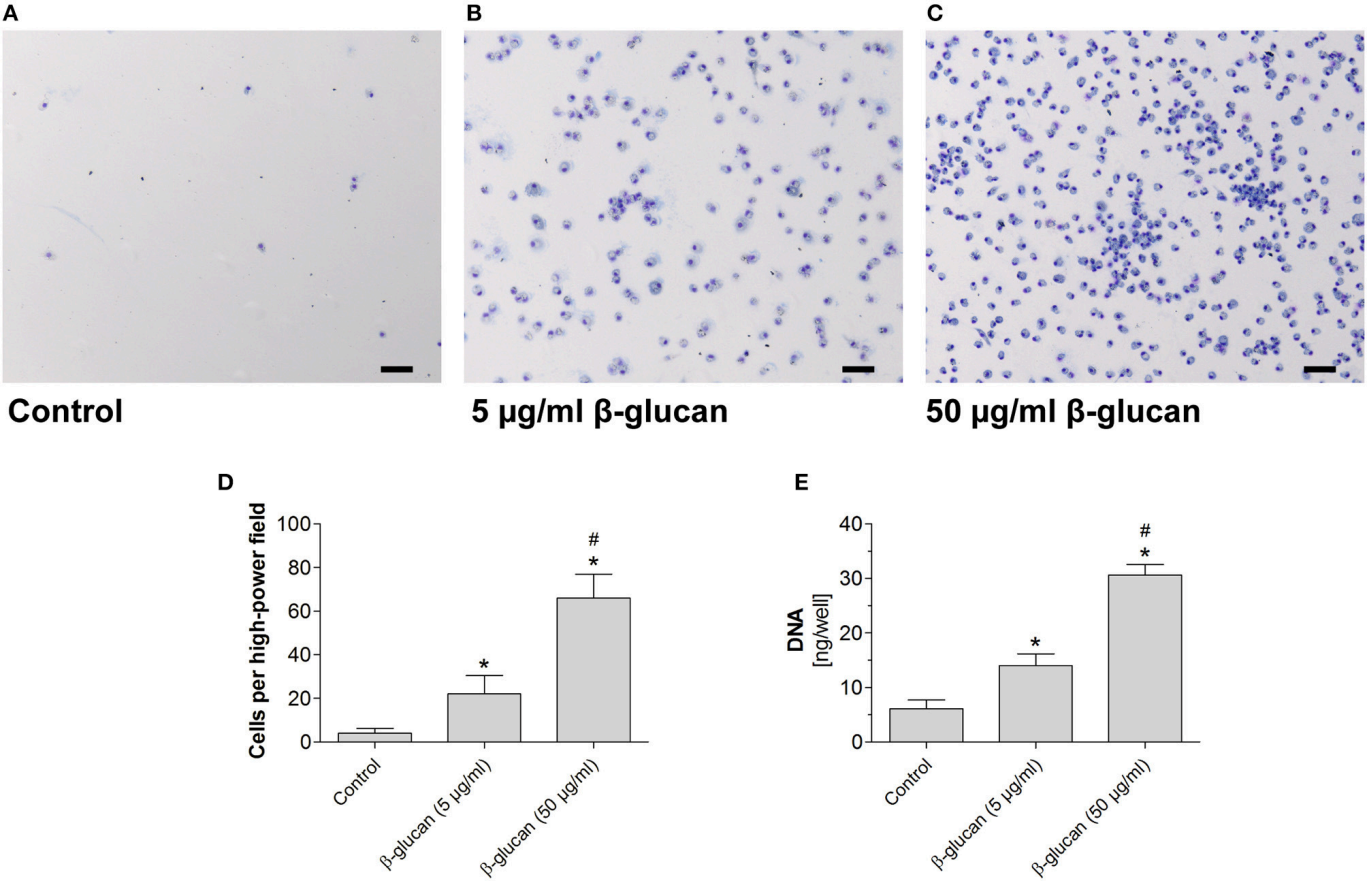
Transient treatment of monocytes with β-glucan increases long-term survival. Human monocytes were left untreated (control) or stimulated with β-glucan (5 μg/ml or 50 μg/ml) for 24 h, followed by washing and 6 further resting days. **(A–C)** May-Grünwald-Giemsa staining of cultured cells after 7 days. The scale bar indicates 100 μm. **(D,E)** The number of surviving cells was evaluated as cell count per high-power field and DNA content per well. Values are means ± SEM from seven **(D)** or four **(E)** independent experiments, ^*^p < 0.05 compared to control, *#p* < 0.05 compared to 5 μg/ml β-glucan.

To test whether the early inhibition of apoptosis induced by transient β-glucan treatment had long-lasting effects, we compared control monocytes with β-glucan-primed cells after a further culture period of 6 days. Only a small number of control monocytes incubated with serum-containing medium survived and differentiated into monocyte-derived cells. Transient treatment with β-glucan resulted in an enhanced long-term survival of monocyte-derived cells as reflected by a higher cell number and an enhanced DNA content per well (Figures 3A–E). The effect was observed with 5 μg/ml and more pronounced with 50 μg/ml β-glucan.

### β-glucan increases cell size and granularity

**Figure 4:**
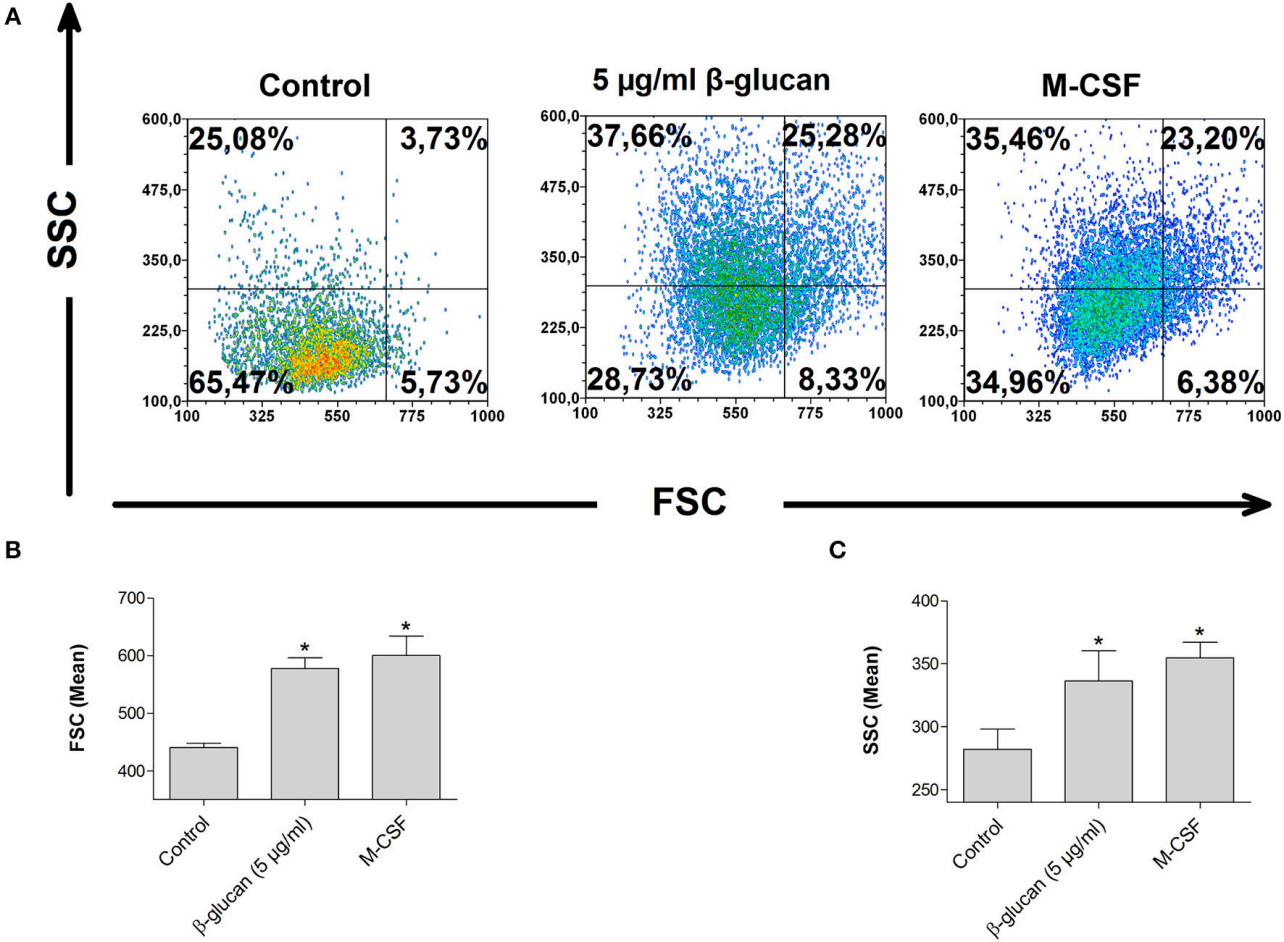
β-Glucan increases cell size and granularity. Human monocytes were left untreated (control) or stimulated with β-glucan (5 μg/ml) or M-CSF (50 ng/ml) for 48 h. All viable cells (PI-/Ann-) were analyzed by flow cytometry using forward scatter (FSC) and sideward scatter (SSC). Representative dot plots **(A)** and means ± SEM from five independent experiments are shown **(B,C)**, ^*^p < 0.05 compared to control.

Monocyte differentiation is characterized by morphological alterations leading to large granulated cells (15). We therefore assessed cell size and granularity of monocytes after 48 h incubation with β-glucan by flow cytometry. Both parameters were significantly increased after treatment with β-glucan. The effect of β-glucan was similar to differentiation mediated by M-CSF (Figures 4A–C), an established monocyte growth factor.

### β-glucan increases metabolic activity

**Figure 5:**
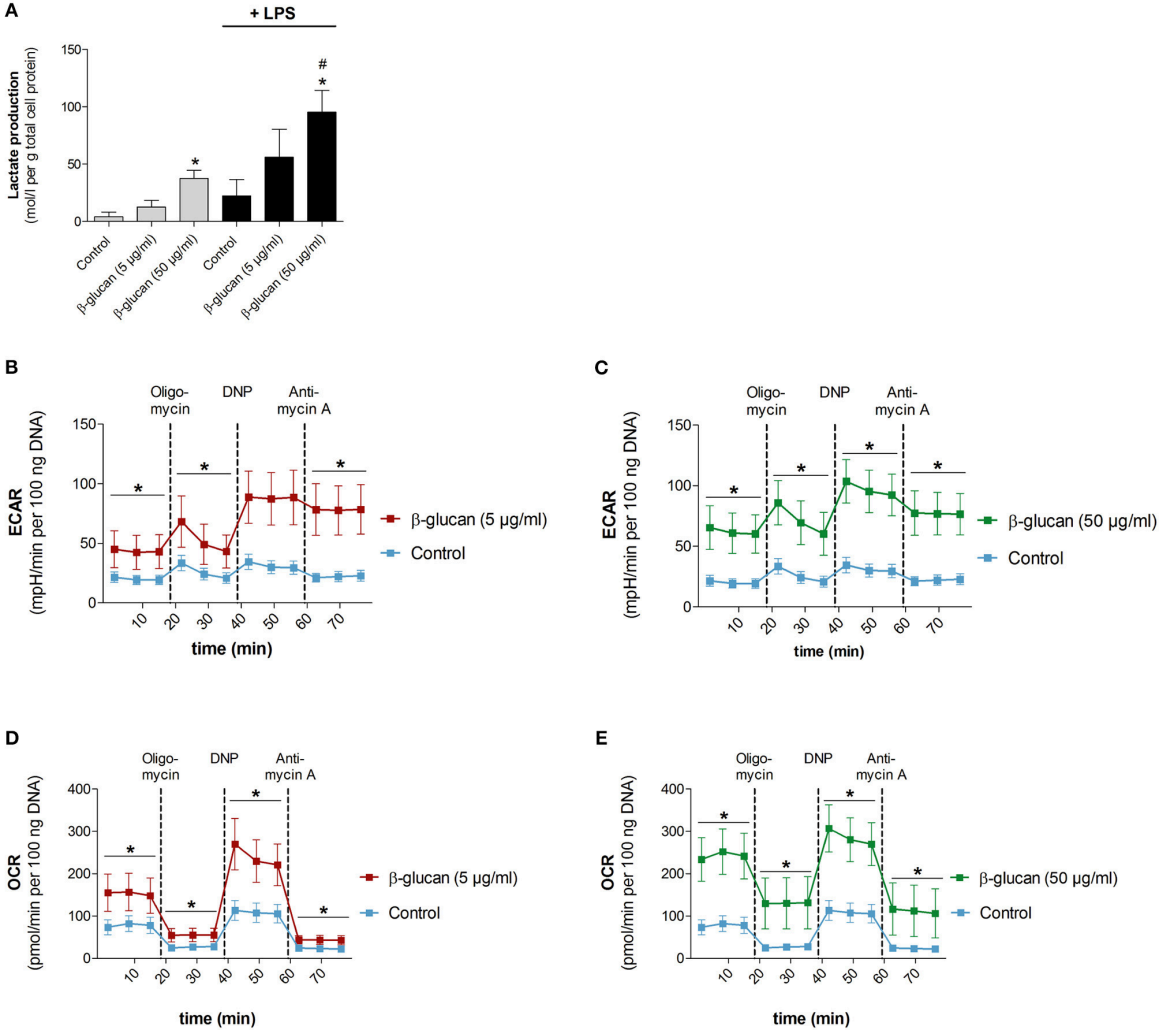
β-Glucan increases metabolic activity. Human monocytes were left untreated (control) or incubated with β-glucan (5 μg/ml or 50 μg/ml) for 24 h, followed by washing and a resting period for 6 further days. **(A)** Medium was exchanged on day 6 and cells were stimulated with 10 ng/ml LPS (black bars) or left untreated (gray bars). Lactate levels of cell supernatants were determined 24 h later (day 7). **(B–E)** extracellular acidification rate (ECAR) and oxygen consumption rate (OCR) were determined at baseline, after oligomycin treatment (inhibition of mitochondrial ATP synthase), after 2,4-dinitrophenol (DNP, mitochondrial uncoupling agent) and after antimycin A [inhibition of electron transport chain (ETC) complex III]. All ECAR and OCR results were normalized to the DNA content of cells. Values are means ± SEM from five **(A)** or four **(B–E)** independent experiments, ^*^p < 0.05 compared to control, *#p* < 0.05 compared to 5 μg/ml β-glucan.

**Figure 6:**
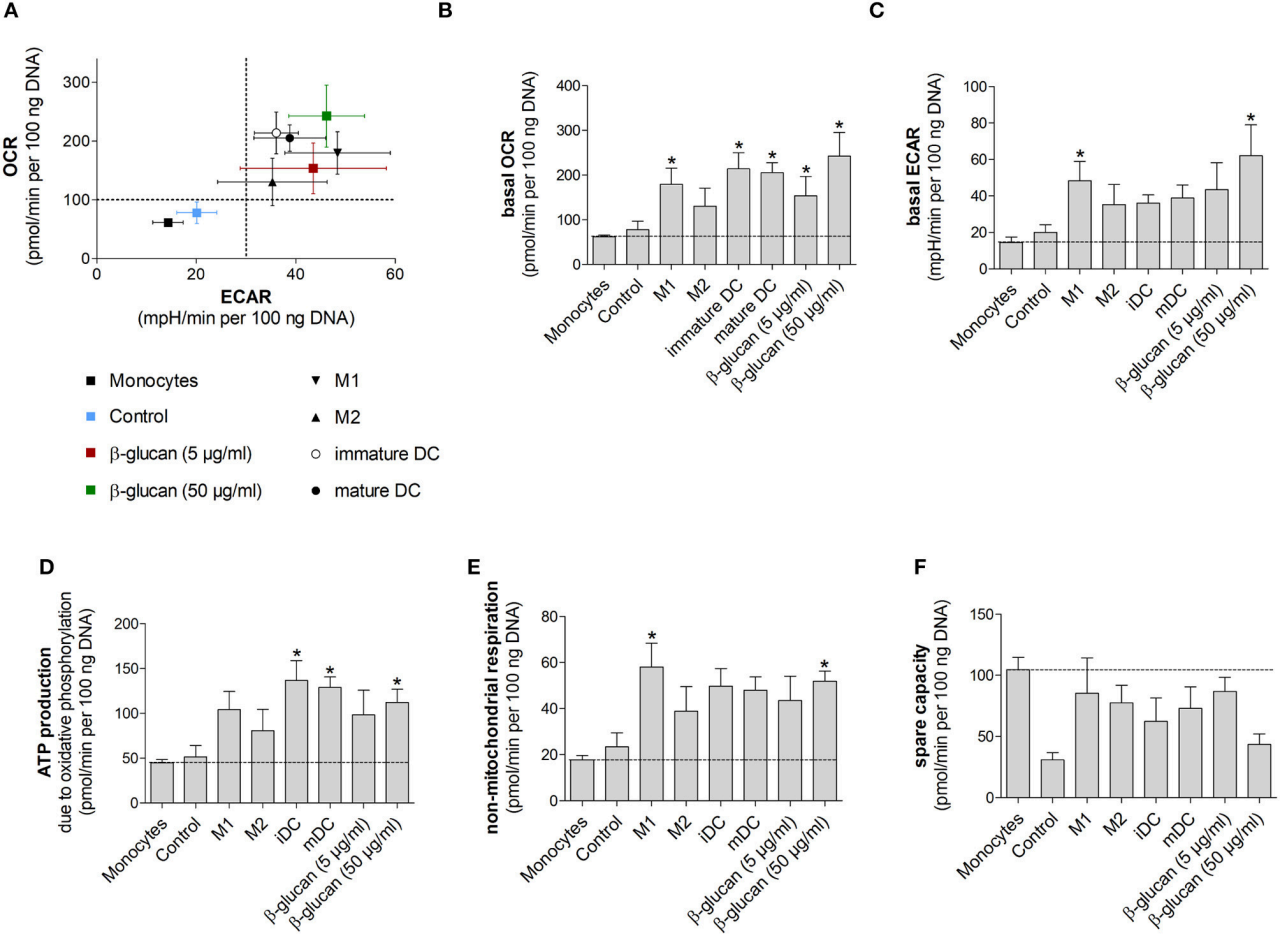
β-Glucan-induced metabolic phenotype resembles differentiated monocyte-derived cells. **(A–F)** Human monocytes prepared from one donor were either directly analyzed for metabolic data (monocytes) or were differentiated to monocyte-derived cells. To obtain β-glucan-differentiated cells, monocytes were incubated for 24 h with β-glucan (5 μg/ml or 50 μg/ml), followed by washing and resting for 6 further days. M1-like macrophages (M1) were differentiated with GM-CSF and polarized with LPS + IFNγ. M2-like macrophages (M2) were differentiated with M-CSF and polarized with IL-4. Dendritic cells (DC) were differentiated with GM-CSF + IL-4 (immature DC) and were matured with LPS (mature DC). Monocytes left untreated for 7 days (control) served as additional control. Extracellular acidification rate (ECAR) and oxygen consumption rate (OCR) were determined at baseline and after treatment with oligomycin [inhibition of complex V of the electron transport chain (ETC)] plus 2,4-dinitrophenol (DNP, mitochondrial uncoupling agent). **(A–C)** OCR and ECAR are shown under baseline conditions. **(D–F)** Mitochondrial ATP production, non-mitochondrial respiration and spare capacity were calculated from OCR. All ECAR and OCR results were normalized to the DNA content of cells. Values are means ± SEM from four independent experiments. *p < 0.05 compared to monocytes.

To test whether the β-glucan-induced differentiation is accompanied by metabolic alterations we analyzed metabolic parameters 6 days after transient stimulation with β-glucan and compared them with control cells incubated for the same time. Figure 5A shows that β-glucan-treated cells produced more lactate indicating an increase in aerobic glycolysis, which was more pronounced upon stimulation with LPS (10 ng/ml, 24 h). Metabolic activity was also monitored using a Seahorse extracellular flux analyzer. In line with the higher lactate production we found that β-glucan-treated cells had a higher extracellular acidification rate (ECAR) already at basal levels and more significant after subsequent treatment with oligomycin and DNP indicating an increased glycolytic activity (Figures 5B–C). In addition, basal and DNP-uncoupled oxygen consumption rates (OCR) were significantly enhanced demonstrating that oxidative metabolism was also intensified after transient β-glucan treatment (Figures 5D,E). Interestingly, while the maximum OCR and ECAR values monitored after DNP treatment were comparable after priming with 5 or 50 μg/ml β-glucan, the increase of basal metabolic activity was more pronounced with the higher concentration of the compound (Figures 5B–E). Accordingly, the glycolytic and mitochondrial spare capacities representing the differences between DNP-uncoupled and basal ECAR and OCR, respectively, were lower in cells treated with 50 μg/ml β-glucan. Thus, cells differentiated with higher doses of β-glucan were metabolically more active using a larger part of their metabolic reserve, while the metabolic potential was not different between cells primed with 5 μg/ml or 50 μg/ml β-glucan (see also Figure 6F).

### β-glucan-induced metabolic phenotype resembles differentiated monocyte-derived cells

We next performed Seahorse analyses of β-glucan-primed cells, their respective controls, freshly isolated monocytes and monocyte-derived cells differentiated into M1- or M2-like macrophages or dendritic cells. Dendritic cells were included to compare our results to previous studies, in which dendritic cell markers after β-glucan treatment were investigated (10, 16). All cells used in an individual experiment were derived from the same donor. Figure 6A shows scatter plots of basal ECAR and OCR of all investigated cells as an overview, while Figures 6B–F demonstrate individual OCRs, ECARs or parameters calculated from OCRs. While unstimulated cells, i.e., freshly prepared monocytes and cells incubated for 7 days without any treatment (control), displayed a low metabolic activity, cells differentiated by β-glucan or monocyte-directed growth factors exhibited higher oxygen consumption and glycolysis (Figures 6A–C). In line with this, ATP production was enhanced in monocyte-derived macrophages, dendritic cells and β-glucan-primed cells while freshly prepared monocytes and control cells showed lower values of ATP production (Figure 6D). Similar differences were observed for non-mitochondrial respiration (Figure 6E). The higher metabolic activity in β-glucan-treated and differentiated cells as well as the lower reserve capacity observed in parallel (Figure 6F) point to an increased cellular energy demand upon differentiation. Together, these data indicate that differentiated cells utilize both energy-producing pathways — glycolysis and mitochondrial respiration — and that β-glucan induces a metabolic phenotype similar to the one induced by established monocyte differentiation factors.

### β-glucan differentiates monocytes into macrophage-like cells

**Figure 7:**
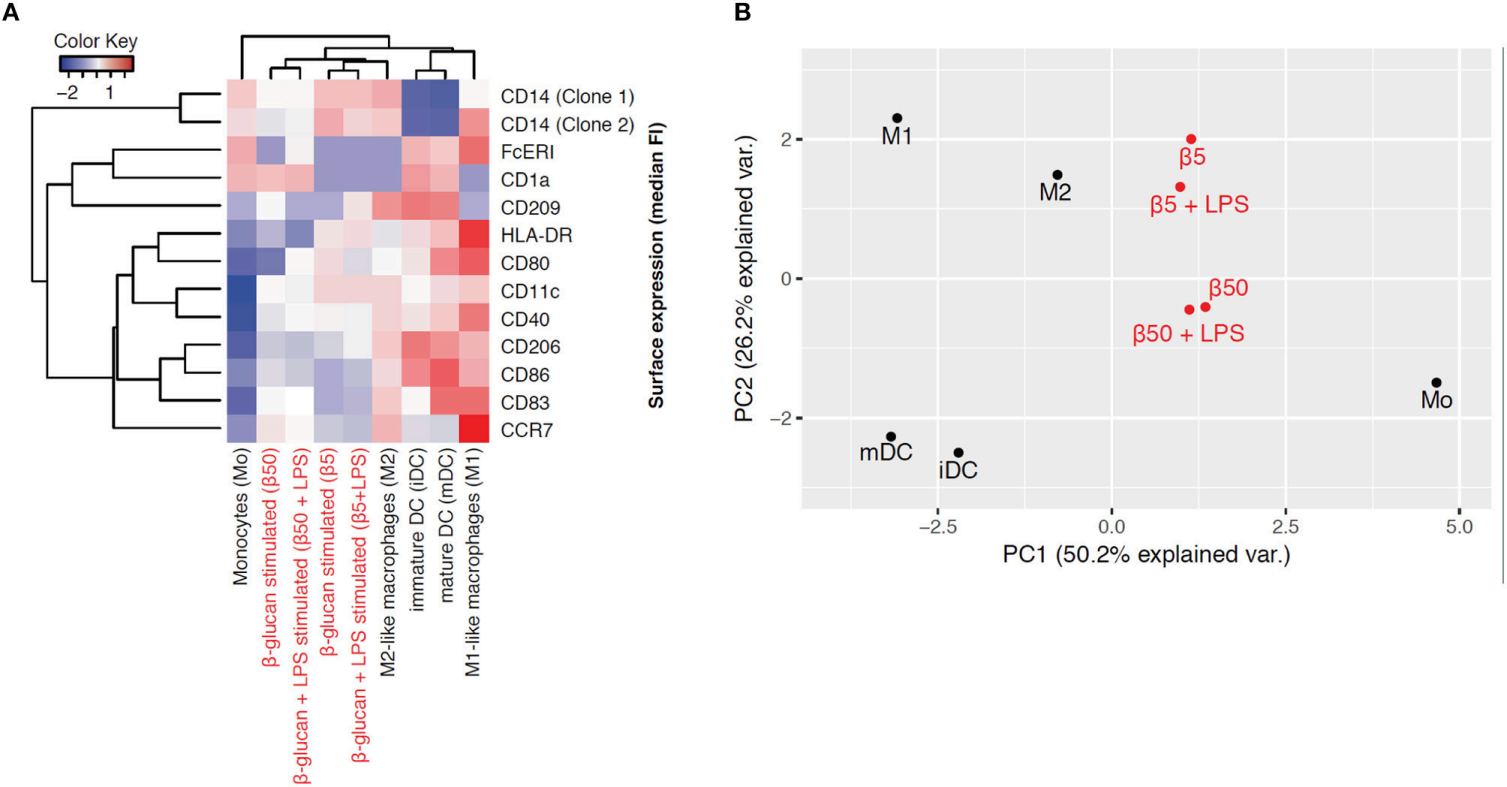
β-Glucan differentiates monocytes into macrophage-like cells. **(A-B)** Human monocytes (Mo) from one donor were either analyzed for expression of cell surface markers by FACS or differentiated for 7 days to monocyte-derived cells. To obtain β-glucan-differentiated cells (β5, β50), monocytes were incubated for 24 h with 5 μg/ml (β5) or 50 μg/ml β-glucan (β50), followed by washing and resting for 5 further days. On day 6, cells were stimulated with 10 ng/ml LPS or left untreated. M1-like macrophages (M1) were differentiated with GM-CSF and polarized with LPS + IFNγ. M2-like macrophages (M2) were differentiated with M-CSF and polarized with IL-4. Dendritic cells were differentiated with GM-CSF + IL-4 (immature dendritic cells, iDC) and were matured with LPS (mature dendritic cells, mDC). Heat map displaying the z-normalized median fluorescence intensity (MFI) of surface expression markers **(A)** and principal component analysis **(B)** of isolated monocytes and monocyte-derived cells are shown. One representative experiment out of 2.

To explore the phenotype of monocytes 6 days after transient β-glucan stimulation in more detail, we analyzed the expression of several surface markers classically related to monocyte differentiation. For comparison, freshly prepared monocytes and monocytes from the same donor differentiated into pro-inflammatory macrophages (M1-like), resolving macrophages (M2-like), immature dendritic cells (iDC), and mature dendritic cells (mDC) by established pretreatments were examined. These phenotypes have previously been regarded as major subsets of monocyte-derived cells but are now considered to be part of a dynamic range of various phenotypes. Figure 7A shows a heat map displaying the mean fluorescence intensity (MFI) of surface expression markers. Treatment of monocytes with β-glucan led to differentiation into macrophages with a distinct marker profile, which was characterized by lower expression of HLA-DR and co-stimulatory molecules compared to monocyte-derived M1 macrophages and moDCs. This expression profile was close to the profile of monocyte-derived M2 macrophages, particularly after treatment with 5 μg/ml β-glucan (Figure 7A). Unsupervised principal component analysis of isolated monocytes and monocyte-derived cells revealed that transient β-glucan treatment induced a macrophage phenotype sharing some traits of M2-like monocyte-derived macrophages (Figure 7B). A secondary treatment of β-glucan-primed cells with LPS did not change their phenotype (Figures 7A,B).

### β-glucan-differentiated cells release pro- and anti-inflammatory cytokines and kill live bacteria

**Figure 8:**
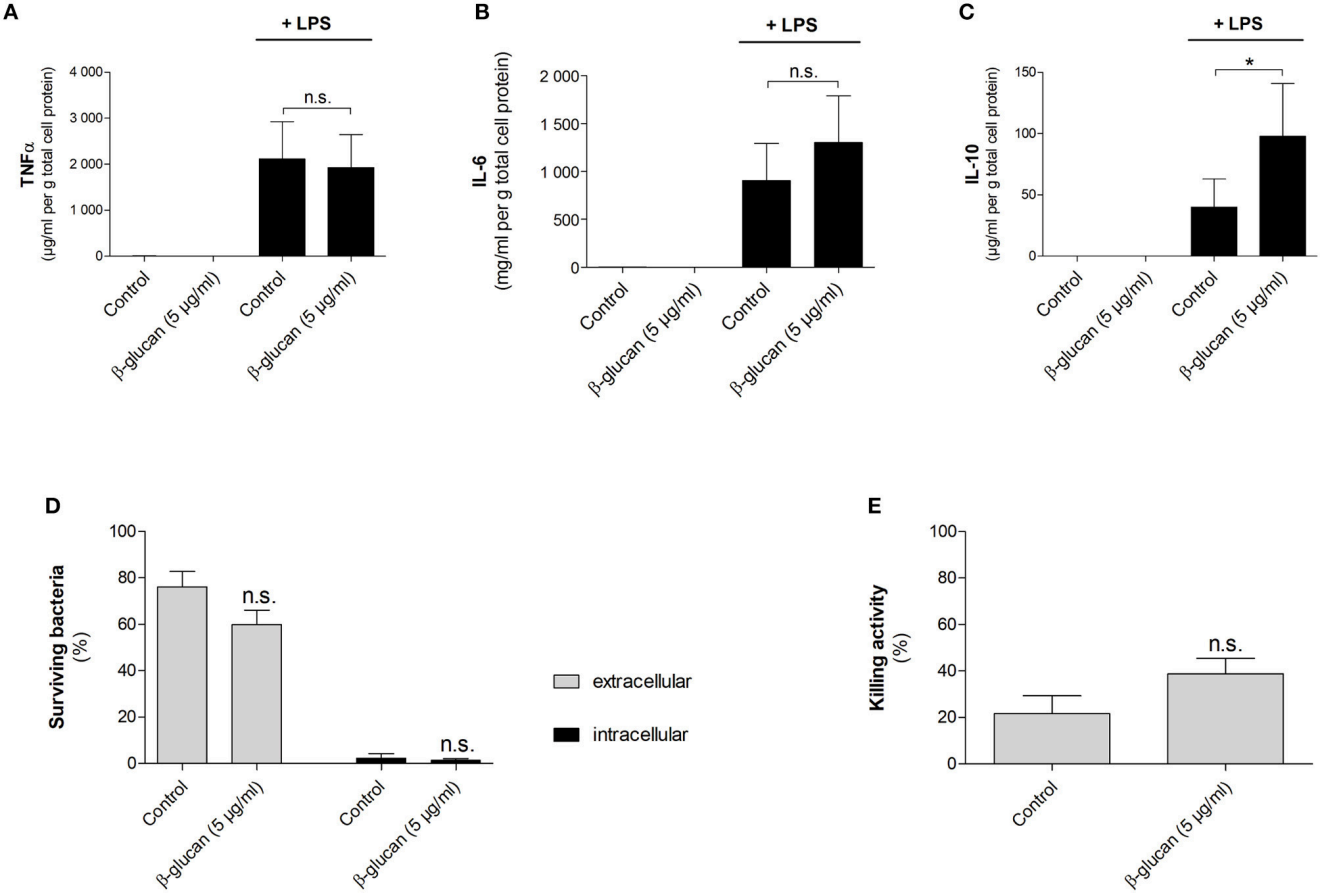
β-Glucan-differentiated cells release pro- and anti-inflammatory cytokines and kill live bacteria. Human monocytes were left untreated (control) or stimulated with 5 μg/ml β-glucan for 24 h, followed by washing and resting for 5 further days. **(A–C)** Medium was exchanged on day 6. Cells were stimulated with 10 ng/ml LPS for 24 h or left untreated. Cytokine release in supernatants was determined by cytometric bead array and normalized to cell proteins. Values are means ± SEM from six independent experiments. **(D,E)** Cells were detached and reseeded to 6-well plates on day 6. After 24 h, cell numbers were determined and infection with *S. aureus* was performed at a MOI of 5 for 90 min. Extra- and intracellular bacteria were quantified by CFU measurements. The killing activity was calculated as difference between the initial number of bacteria and the number of surviving extra- and intracellular bacteria in % of the initial inoculum. Values are means ± SEM from six independent experiments, n.s. not significant, ^*^p < 0.05.

To characterize macrophage-like cells induced by transient β-glucan treatment functionally, we first measured LPS-induced cytokine release 6 days after priming. Neither β-glucan-stimulated cells nor control cells produced TNFα, IL-6, or IL-10 spontaneously, but both cell populations responded to 24 h stimulation with LPS with an adequate cytokine release (Figures 8A–C). While LPS-induced TNFα secretion was comparable between control cells and β-glucan-treated cells, IL-6 release was slightly enhanced. However, a significantly increased production of IL-10, a cytokine typically secreted by M2-like macrophages, was observed after β-glucan priming. Secondly, we investigated the bactericidal activity of cells transiently treated with β-glucan and control cells after incubation with live *S. aureus*. Figures 8D–E show that both cell populations were able to kill bacteria but bacterial counts in supernatants and cells were comparable between control cells and β-glucan-primed cells. Accordingly, the killing activity calculated as difference between initially added and surviving bacteria did not show a significant difference between the two conditions. These data confirm on a functional level that β-glucan differentiates monocytes into a distinct subpopulation with characteristics typically assigned to classical and to alternatively activated monocyte-derived cells.

## Discussion

Our study employed a previously published model to investigate immunomodulatory effects of β-glucan on human monocytes (6, 17, 18). We show that transient treatment of monocytes with β-glucan inhibits spontaneous apoptosis and stimulates long-term differentiation into a specific subset of immune competent macrophages. These cells are characterized by enhanced metabolic activity and a distinct surface marker profile resembling traits of M2-like macrophages. β-Glucan-differentiated cells have the ability to clear bacteria and to respond to LPS with the release of pro- and anti-inflammatory cytokines suggesting that they may play a role in controlling inflammatory processes.

Monocytes are known to undergo spontaneous apoptosis in the absence of specific differentiation factors (13, 19). In line with this, we observed that monocytes kept in cell culture medium rapidly developed signs of apoptosis such as activation of caspase 3 and cleavage of PARP, a major target of caspase 3. Accordingly, binding of annexin V was enhanced and the combined staining of annexin V and PI revealed that only 62.5% of all cells were viable 48 h after isolation. When monocytes were treated with β-glucan, caspase-3 activation and PARP cleavage were inhibited and more than 85% of cells survived. This effect was comparable to that of M-CSF, which is known to trigger survival signals such as the PI3K/Akt pathway (20). Since an enhanced expression of M-CSF in β-glucan-stimulated human monocytes was described previously (21) and confirmed in our study, we hypothesized that M-CSF mediates the anti-apoptotic effect of β-glucan in an auto- or paracrine manner. However, pretreatment of monocytes with an M-CSF-receptor inhibitor did not alter the anti-apoptotic effect of β-glucan while it clearly prevented the beneficial effect of M-CSF. These data suggest that M-CSF was not responsible for the anti-apoptotic activity of β-glucan. Rather, β-glucan may directly activate the PI3K/Akt pathway via its receptor dectin-1, which has been described recently (6). Importantly, inhibition of apoptosis by β-glucan led to enhanced long-term survival and allowed differentiation of monocytes during the subsequent cultivation. After 6 days, up to 16-fold higher cell numbers were observed compared to control cells (Figure 3). These data are in line with previous data showing that β-glucan training led to an enhanced survival of cultured murine and human monocytes (10, 22). An increased survival rate together with stimulation of myelopoiesis may lead to increased numbers of circulating monocytes seen *in vivo* (23, 24). In line with this, the role of myelopoiesis as an integral component of trained (innate) immunity has recently been highlighted (2, 5).

In addition to survival, we assessed further key features of monocyte-to-macrophage-differentiation such as morphology and metabolism (15, 25). The induction of differentiation is characterized by an increase in cell size from small monocytes (7–8 μm) to large monocyte-derived cells (15–20 μm and more) with an increased cytoplasmic complexity (15). Cells primed with β-glucan showed significant increases in size and granularity compared to untreated monocytes thus indicating that these cells underwent differentiation (Figure 4). The morphological changes were similar to the effects induced by M-CSF, an established differentiation stimulus.

β-Glucan-trained macrophages were also metabolically different from naïve monocytes and control cells as demonstrated by increased oxygen consumption, enhanced extracellular acidification rate, a higher ATP production rate and increased lactate production (Figures 5, 6). Metabolic changes in immune cells, especially high aerobic glycolysis, are part of the inflammatory response by ensuring energy homeostasis, limiting damage caused by reactive oxygen species and allowing the expression of inflammatory genes (26). Earlier studies have shown that β-glucan treatment led to increased glycolysis, which was seen as metabolic basis for trained immunity (3, 6, 18). Mechanistically, increased glycolytic flux was related to increased mTOR activation via a dectin-1/Akt/HIF1α pathway and to epigenetic and transcriptional modifications (6, 7). More recent studies identified fumarate, which accumulates due to enhanced glutaminolysis, and mevalonate in β-glucan primed macrophages as a link between immunometabolism and epigenetic regulation (3, 18). Fumarate not only supports histone modifications such as H3K4me3 by inhibiting the histone demethylase KDM5 but also prevents proteasomal degradation of HIF1 thereby contributing to enhanced expression of glycolytic genes (27, 28).

Previous studies interpreted metabolic effects of β-glucan as a metabolic shift toward glycolysis, the more so that oxygen consumption and the capacity of the mitochondrial electron transport chain seemed to be reduced at the same time (6). Our study confirms an increased glycolytic flux in β-glucan-primed macrophages but shows increased oxygen consumption in parallel. The influence of β-glucan on cell metabolism appeared to be dose-dependent since spare capacities were lower when cells were treated with 50 compared to 5 μg/ml, indicating that cells treated with the higher dose β-glucan were metabolically more active. Our results are in line with data on monocytes stimulated with other inducers of trained immunity such as heat-killed *C. albicans* yeast or BCG (29, 30). Increased oxygen consumption could, for instance, be related to β-glucan-triggered glutaminolysis (18), whose metabolites fuel the tricarboxylic acid cycle and, in turn, mitochondrial oxidative phosphorylation. Not only β-glucan-primed macrophages but all monocyte-derived macrophages investigated in our study exhibited a metabolically highly active phenotype with both glycolysis and oxygen consumption being enhanced (Figure 6). These data suggest that metabolic reprogramming of activated macrophages may be more complex as previously suggested which requires further detailed studies.

To better define differentiation, we performed a phenotyping analysis based on different surface markers and compared β-glucan-primed cells with specific subsets of monocyte-derived macrophages and dendritic cells. We found that β-glucan priming of monocytes triggered their differentiation into macrophages with a distinct phenotype, which was closest to M2-like macrophages and more different from monocyte-derived dendritic cells or M1-like macrophages. Previous studies had suggested that β-glucan differentiates murine and human monocytes away from dendritic cell-like phenotypes or toward dysfunctional dendritic cells, respectively (10, 16). Our data underline that the initiation of monocyte differentiation *in vitro* is not only restricted to cytokines and growth factors such as M-CSF and GM-CSF but can also be induced by PAMPs such as β-glucan. In line with this, the presence of PAMPs or danger-associated molecular patterns (DAMPs, e.g., oxidized LDL, cell debris, hemin) has been shown to initiate monocyte differentiation (31–35). Moreover, several bacterial, fungal, and viral ligands have been shown to induce functional reprogramming of monocytes although a detailed phenotyping of cells has not been performed in these studies (10, 36). Our study confirms that the transcriptional repertoire of monocytes is much broader than the historical classification system into proinflammatory M1 and anti-inflammatory M2 macrophages had been suggested (37). Depending on the local tissue environment and the varying presence of cytokines, microbes, microbial products and activated lymphocytes, various subsets of differentiated macrophages can develop, which are heterogeneous and overlapping with respect to surface markers and functions and may even dynamically change from one phenotype to another (38).

β-Glucan-differentiated macrophages were able to kill bacteria although this was not different from control cells. As expected, they responded to LPS stimulation by releasing pro- and anti-inflammatory cytokines. However, when controlled for cell survival, only a trend toward an increased LPS-induced IL-6 release was seen in β-glucan-primed macrophages as compared to controls, while an enhanced TNFα secretion was not observed in contrast to previous reports (6, 17). These observations agree with a recent study in human and murine monocytes (10) and point to increased survival and subsequent differentiation of macrophages as major components of β-glucan priming. This does not exclude that an enhanced secretion of inflammatory cytokines may occur under distinct microenvironmental conditions. During infections patients might profit from both an increased number of immune cells (due to enhanced myelopoiesis and survival rate) and an improved individual cell function. Interestingly, β-glucan-primed macrophages release IL-10, an anti-inflammatory cytokine, which is a hallmark of M2-like macrophages (39, 40). IL-10 might exert beneficial effects during acute infection by preventing exacerbated inflammation and tissue damage. Its secretion by β-glucan-differentiated cells may modulate the immune response, which points to a possible role of this subset in limiting pathology.

Together, the data of the current study demonstrate that β-glucan priming leads to an enhanced survival and differentiation into a distinct subset of monocyte-derived macrophages, which are highly metabolically active, able to kill bacteria and to release pro- and anti-inflammatory cytokines. Thus, β-glucan-differentiated macrophages may not only be involved in pathogen control but also in limiting pathological alterations in tissues.

## Ethics statement

This study was carried out in accordance with the recommendations of the Ethical Committee of the Jena University Hospital. All subjects gave written informed consent in accordance with the Declaration of Helsinki. The protocol was approved by the Ethical Committee of the Jena University Hospital (ethic vote: 4819-06/16).

## Author contributions

JL, SW, and RH designed the research. DW provided the *C. albicans* derived β-glucan. JL, FS, CM, SG, SS, TB, MK, and AM acquired the experimental data. RB performed the statistical analysis. JL and RH drafted the manuscript. All authors critically revised the manuscript.

### Conflict of interest statement

The authors declare that the research was conducted in the absence of any commercial or financial relationships that could be construed as a potential conflict of interest. The reviewer SH and handling Editor declared their shared affiliation at the time of review.

## Abbreviations

DAMP: danger-associated molecular pattern
DC: dendritic cells
DNP, 2: 4-dinitrophenol
ECAR: extracellular acidification rate
ETC: electron transport chain
GM-CSF: granulocyte-macrophage colony-stimulating factor
iDC: immature dendritic cells
M1: M1-like macrophages
M2: M2-like macrophages
M-CSF: macrophage colony-stimulating factor
mDC: mature dendritic cells
OCR: oxygen consumption rate
PAMP: pathogen-associated molecular pattern.
